# Supplementary material for: UBL3 Interacts with Alpha-Synuclein in Cells and the Interaction Is Downregulated by the EGFR Pathway Inhibitor Osimertinib
Source: Biomedicines. 2023 Jun 10;11(6):1685. doi: 10.3390/biomedicines11061685 (PMC10295865; doi:10.3390/biomedicines11061685)
Supplement: Supplementary file 1 [file biomedicines-11-01685-s001.zip › biomedicines-2401235-supplementary.pdf]

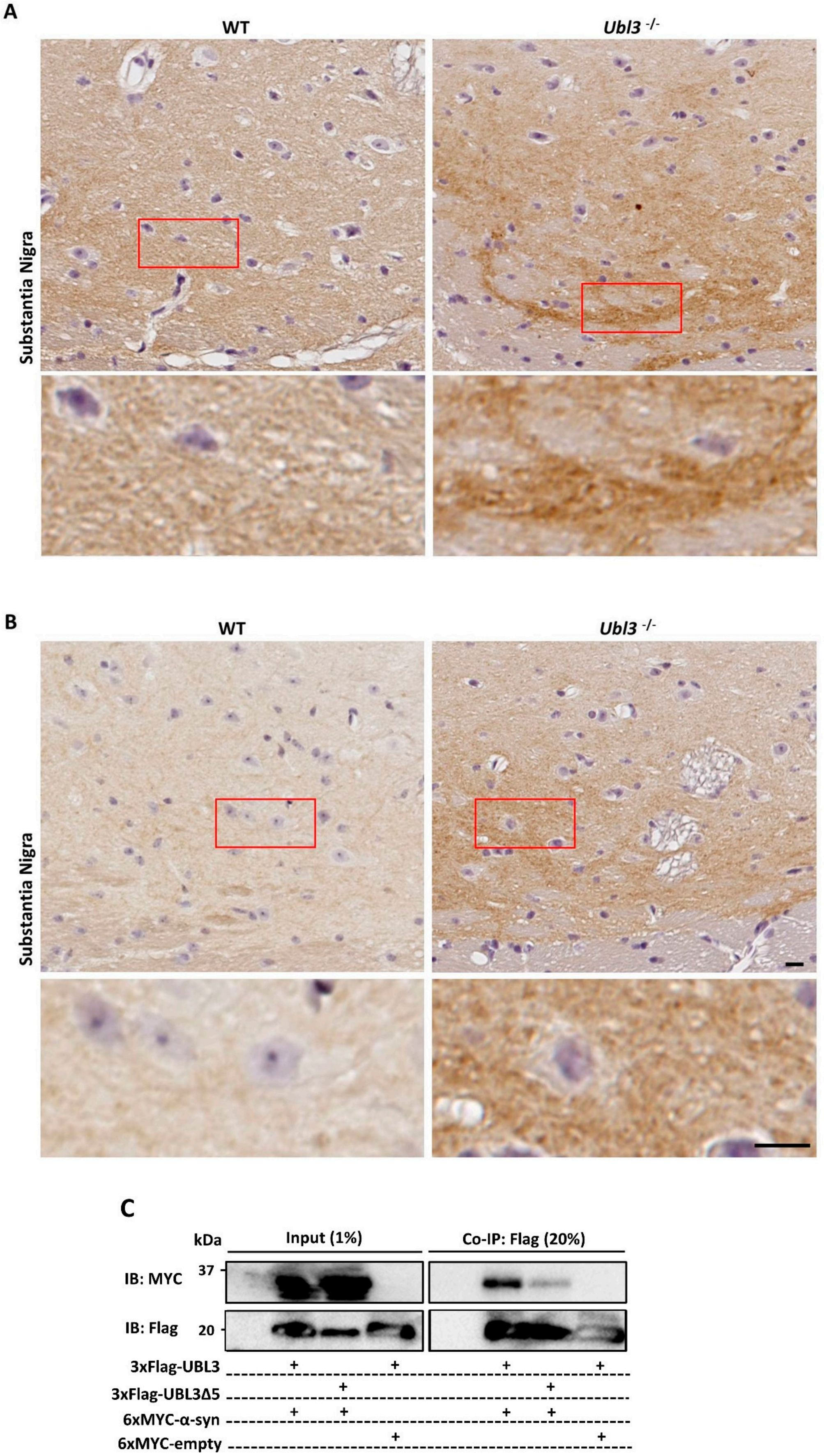

A
WT
Ubl3 -/-
Substantia Nigra
B
WT
Ubl3 -/-
Substantia Nigra
C
kDa
Input (1%)
Co-IP: Flag (20%)
IB: MYC
37
IB: Flag
20
3xFlag-UBL3
3xFlag-UBL3Δ5
6xMYC-α-syn
6xMYC-empty

Supplementary Figure S1. Reproduced IHC staining and co-immunoprecipitation (Co-IP results). (A) (B) Representative images of reproduced immunocytochemistry staining of p-S-129 α-syn in the substantia nigra of WT and Ubl3-/- mice. Scale bars: 10 μm. (C) Reproduced result of co-immunoprecipitated 3xFlag-UBL3 and 3xFlag-UBL3Δ5 interact with 6xMYC-α-syn. The input lanes are 1% of the sample prior to Co-IP, and the Co-IP lanes are 20% of the Co-IP products. WT: wild type. Ubl3-/-: Ubl3 knock out.

Supplementary Table S1. Drug list

Drug	Supplier	Product ID
Apomorphine	Wako Bio	013-18323
Aniracetam	Combi-Blocks	OR-2762
Chlorpromazine	TCI	C2481
Chloroquine Diphosphate	Wako Bio	038-17971
Curcumin	Wako Bio	038-04921
Caffeic acid	Cayman Chemical Co.	70602
Cisplatin	Wako Bio	033-20091
Docetaxel	LKT Labs, Inc	D5709-10mg
Donepezil	Combi-Blocks	ST-7783
Ergoloid	Cayman Chemical Co.	24095
Erlotinib	Med Chem Express	HY-50896
Farnesol	Wako Bio	064-03941
Famotidine	Wako Bio	066-06701
Gefitinib	Wako Bio	078-06561
Gemcitabine	Combi-Blocks	QA-8591
Haloperidol	Wako Bio	084-04261
Memantine hydrochloride	Combi-Blocks	OR-1164
Melatonin	Wako Bio	139-17111
Mannitol	Wako Bio	139-00842
Methylcobalamin	Wako Bio	138-14261
Medetomidine Hydrochloride	Wako Bio	139-17471
Osimertinib	LC Laboratories	O-7200
Pemetrexed	Combi-Blocks	QB-9701
Pravastatin	Cayman Chemical Co.	10010342
Rivastigmine	Combi-Blocks	QC-5226
Simvastatin	Fluorochem Ltd.	M03997
Sulfasalazine	MP Biomedicals, Inc.	191144
Safinamide	Cayman Chemical Co.	S1472
Scutellarin	Cayman Chemical Co.	27461
Trifluoperazine	TCI	T2849
Tetrabenazine	Toronto Research Chemicals Inc	T284000
Vitamin D2	LKT Labs, Inc	V3476
